# Complete mitochondrial genome of the muskrat (*Ondatra zibethicus*) and its unique phylogenetic position estimated in *Cricetidae*

**DOI:** 10.1080/23802359.2017.1390402

**Published:** 2018-03-01

**Authors:** Haiping Zhao, Xiaoyan Qi, Chunyi Li

**Affiliations:** a Institute of Special Wild Economic Animals and Plants, Chinese Academy of Agricultural Sciences, Changchun, China;; b State Key Laboratory for Molecular Biology of Special Economic Animals, Changchun, China

**Keywords:** Complete mitochondrial genome, *Cricetidae*, *Ondatra zibethicus*, phylogenetic analysis

## Abstract

*Ondatra zibethicus* belongs to the genera *Ondatra* within the subfamily *Arvicolinae*, its complete mitochondrial genome is 16,348 bp in length, containing 13 protein-coding genes, 22 tRNA genes, 12S rRNA gene and 16S rRNA gene as other Cricetidae species. Results of phylogenetic analysis showed that *Ondatra* had unique phylogenetic position estimated in *Cricetidae* between *Myodes*, *Microtus*, *Wiedomys*, *Akodon*, *Cricetulus*, *Onychomys* and other genera. This study revealed the evolutionary status of *O. zibethicus* in *Ondatra* at the molecular level. The mitochondrial genome would be a significant supplement for the *O. zibethicus* genetic background analysis and experimental animalization.

Muskrat (*Ondatra zibethicus*), the only species in genus *Ondatra* and the largest species in subfamily *Arvicolinae*, is a medium-sized semiaquatic rodent found in North America, Europe and Asia (Gintarė Skyrienė 2012). The muskrats are found in wetlands over a wide range of climates and habitats. They feed on cattails and other aquatic vegetation, but they also eat small animals such as crayfish and fish. In some European countries, such as Belgium, France, and the Netherlands, the muskrat is considered an invasive pest (Robertson et al. 2017), as its burrowing damages the dikes and levees on which these low-lying countries depend for protection from flooding. In some part of China, the muskrat is considered a kind of precious domesticated animals (Xu et al. 2006) based on the secreted muskrat perfume.

The muskrat perfume which has a strong typical sweet aroma is secreted through the muskrat gland during April to September annually by male muskrats (Lu et al. 2014; Zhang et al. 2017). Muskrat perfume can be used as the material of producing senior perfume and used as precious medicine material instead of musk. The muskrat was reported as an intermediate host of cestodes, playing roles as carriage of larval *Echinococcus multilocularis* and other cestodes in the Netherlands, Belgium and other countries (Borgsteede et al. 2003; Mathy et al. 2009). So, the mitochondria genome research on *O. zibethicus* has important significance for the *O. zibethicus* genetic background analysis and experimental animalization.

In this study, the muskrat (*O. zibethicus*) was selected from Animal Science Observation and Experiment station of Ministry of Agriculture of China (Jilin Province). The liver tissue of *O. zibethicus* was sampled to extract total genomic DNA, using TIANamp Genomic DNA Kit (Tiangen Biotech Beijing, Co., Ltd., Beijing, China) according to the instructions of the manufacturer. To amplify the whole mtDNA genome, 18 pairs of primers were used for polymerase chain reaction amplifying the mitochondrial genome. The complete mitochondrial genome of *O. zibethicus* was sequenced on ABI3730XL for the first time. Phred/Phrap/Consed software suite was used for further sequence analyses (Gordon 2003). Phylogenetic analysis was performed by applying the maximum-likelihood (ML) method using MEGA7 software (Kumar et al. 2016).

We downloaded all mitochondrial genome sequences of Cricetidae from GenBank. All sequences included 27 species of 14 genera. To unravel the phylogenetic position of muskrat in *Cricetidae*, we reconstructed the ML tree using MEGA7 (Kumar et al. 2016).

The mitochondrial genome of *O. zibethicus* assembled to a 16,348 bp long circular-mapping molecule. The GC content of the genome was 37.95%. This mitochondrial genome-encoded 37 unique conserved genes: two rRNAs, 22 tRNAs, 13 protein-coding genes encoding respiratory proteins, in accordance with other subfamily Cricetidae species (Lu et al. 2017). The genic regions account for 92.65% (15,147 bp) of the entire genome, where 68.1% (11,133 bp) was represented for protein-coding gene (PCGs). This mitochondrial genome sequence was submitted to the GenBank with the accession number KU177045. As the initiation codon, ATT is for NAD3 and NAD5, and other PCGs have ATG. Similarly, TAA is the termination codon for most of the PCGs, and termination codon TAG is used for NAD1 and NAD4.

The phylogenetic tree constructed using the MEGA method, the ML tree, displayed a consistent topology with very high support values (Figure 1). Results of the phylogenetic analysis showed that *Ondatra* had unique phylogenetic position estimated in *Cricetidae* between *Myodes*, *Microtus*, *Wiedomys*, *Akodon*, *Cricetulus*, *Onychomys* and other genera. This study revealed the evolutionary status of *O. zibethicus* in *Ondatra* at the molecular level. The mitochondrial genome would be a significant supplement for the *O. zibethicus* genetic background analysis and experimental animalization.

**Figure 1.**
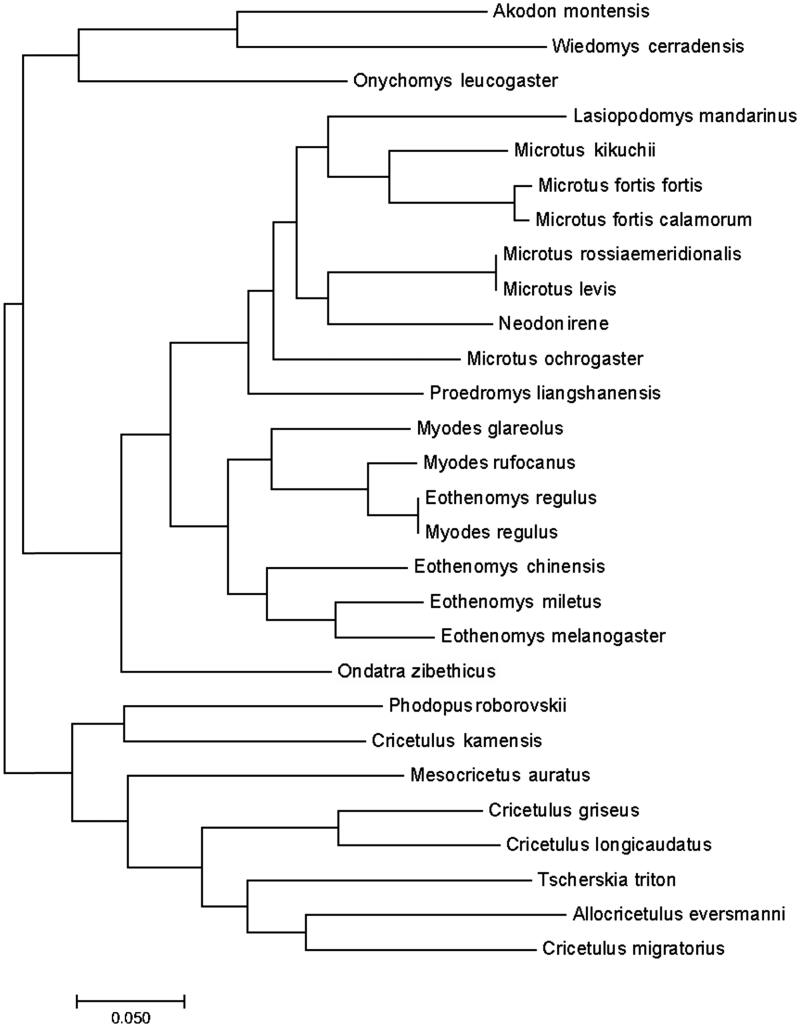
Phylogenetic tree based on the mitochondrial genome of *O. zibethicus* and other 27 species. The GenBank accession number of 27 species are as follows: *Akodon montensis* (NC 025746), *Allocricetulus eversmanni* (NC 027085), *Cricetulus griseus* (NC 007936), *C. kamensis* (NC 024592), *C. longicaudatus* (NC 025330), *C. migratorius* (NC 031802), *Eothenomys chinensis* (NC 013571), *E. melanogaster* (NC 027418), *E. miletus* (NC 030330), *E. regulus* (JN629046), *Lasiopodomys mandarinus* (NC 025283), *Mesocricetus auratus* (NC 013276), *Microtus fortis calamorum* (NC 015243), *Microtus fortis fortis* (NC 015241), *Microtus kikuchii* (NC 003041), *M. levis* (NC 008064), *M. ochrogaster* (NC 027945), *M. rossiaemeridionalis* (DQ015676), *Myodes glareolus* (NC 024538), *M. regulus* (NC 016427), *M. rufocanus* (NC 029477), *Neodon irene* (NC 016055), *Onychomys leucogaster* (NC 029760), *Phodopus roborovskii* (NC 031809), *Proedromys liangshanensis* (NC 013563), *Tscherskia triton* (NC 013068), *Wiedomys cerradensis* (NC 025747).
